# Review of the Permian family Permulidae nomen novum pro Aliculidae Storozhenko, 1997 (Grylloblattida)

**DOI:** 10.3897/zookeys.130.1489

**Published:** 2011-09-24

**Authors:** Daniil S. Aristov, Sergey Yu. Storozhenko

**Affiliations:** 1 Borissiak Paleontological Institute, Russian Academy of Sciences, Moscow, Russia; 2 Institute of Biology and Soil Science, Far East Branch, Russian Academy of Sciences, Vladivostok, Russia

**Keywords:** Grylloblattida, Permulidae, Aliculidae, Ideliidae, Eoblattida, taxonomy, key, new taxa, Permian, Europe, North America

## Abstract

A new replacement name Permulidae **nom. n.** is proposed for the Permian family Aliculidae Storozhenko, 1997 (Insecta: Grylloblattida). A review of Permulidae is given. All genera and species are redescribed and illustrated. A key to genera of Permulidae is given. New taxa of Permulidae are described: *Permula edemskii* **sp. n.**, *Sojanopermula rasnitsyni* **sp. n.**, *Kazanalicula reducta* **gen. et sp. n.**, *Mezenalicula connata* **gen. et sp. n.**, all from the Soyana locality (Middle Permian, Kazanian Stage; Arkhangelsk Region, Russia), and *Mezenalicula conjuncta* **sp. n.** from the Isady locality (Upper Permian, Severodvinian Stage; Vologda Region, Russia). New combinations are proposed: *Permula minor* (Aristov, 2004), **comb. n.** for *Sojanopermula minor* Aristov, 2004; *Permula tshekardensis* (Aristov, 2004), **comb. n.** for *Sojanopermula tshekardensis* Aristov, 2004. A new genus *Acropermula* **gen. n.** (type species: *Permula acra* Kukalová, 1964, from the Lower Permian of Czech Republic) is established in the family Ideliidae. *Neraphidia* Novokshonov & Novokshonova, 1997 is transferred from Aliculidae to the order Eoblattida as a genus of uncertain taxonomic position inside this order.

## Introduction

Family Aliculidae was established for two genera of the Permian Grylloblattida characterized by distinct narrowing of the costal area in the forewing base and by CuA not dividing into CuA1 and CuA2 (Storozhenko 1997). Originally it included the Lower Permian *Alicula lebachensis* Schlechtendal, 1913 from the Saar-Nahe Basin in Germany, *Alicula acra* (Kukalová 1964) from the Obora locality in Czech Republic, the Upper Permian *Alicula asiatica* Storozhenko, 1997 from the Karaungir-II locality in Kazakhstan, and the Middle Permian *Sojanopermula lucida* Storozhenko, 1992 from the Soyana locality in Russia. Aristov (2004a) described in Aliculidae two new species (*Sojanopermula minor* Aristov, 2004, *Sojanopermula tshekardensis* Aristov, 2004) and one monotypic genus (*Neprotembia truncata* Aristov, 2004) from the Lower Permian Tshekarda locality in Russia, and transferred to family Aliculidae the genus *Neraphidia* Novokshonov & Novokshonova, 1997, originally described in the family Protembiidae (Novokshonov and Novokshonova 1997). At the same time he re-examined the holotype of *Alicula asiatica* and placed this species in the genus *Metidelia* Martynov, 1937 of the family Ideliidae (Aristov 2004b). *Neprotembia complicata* Aristov, 2005 was described from the Lower Permian Vorkuta locality in Russia (Rasnitsyn et al. 2005). The genus *Elmopterum* with a single species *Elmopterum rotundum* Béthoux & Beckemeyer, 2007 from the Lower Permian Elmo locality in the USA, originally described as Grylloblattida incertae sedis (Béthoux and Beckemeyer 2007), was placed in Aliculidae by Aristov (2009). Finally *Tshepanichoptera lacera* Aristov, 2008 was described from the Middle Permian Chepanikha locality in Russia (Aristov and Bashkuev 2008). Thus, the composition and conception of family Aliculidae are changed considerably during last ten years and a critical examination of this family is necessary. Moreover, the family name Aliculidae Storozhenko, 1997 was based on *Alicula* Schlechtendal, 1913, a junior homonym of *Alicula* Eichwald, 1830 in Mollusca. So, this generic name must be replaced by the synonymic name *Permula* Handlirsch, 1919, and the family name Aliculidae by Permulidae nom. n.

A review of the family Permulidae nom. n. is given below. Two new genera and five new species are described. The keys to genera and to species of each genus are given based on forewing characters. In addition the taxa previously erroneously included in Permulidae are listed, and a new genus of family Ideliidae is established.

## Material

All studied material including the holotypes of new species is deposited in the Borissiak Paleontological Institute of the Russian Academy of Sciences, Moscow (PIN).

The vein symbols are as follows: **C** costa, **SC** subcosta, **R** stem of radius, **RA** radius anterior, **RS** radius sector (= radius posterior), **M** stem of media, **MA** media anterior, **MP** media posterior, **M5** strong oblique vein between stems of media and cubitus, **Cu** stem of cubitus, **CuA** cubitus anterior, **CuA1** first cubitus anterior (if CuA distinctly forked into branched first and simple second veins), **CuA2** second cubitus anterior, **CuP** cubitus posterior, **A1** first anal, **A2** second anal vein.

## Taxonomy

**Order Grylloblattida Walker, 1914**

**Suborder Protoperlina Brongniart, 1885**

### Family Permulidae

Aristov & Storozhenko nom. n.

http://species-id.net/wiki/Permulidae

#### Type genus.

*Permula* Handlirsch 1919.

Aliculidae Storozhenko, 1997: 8 (type genus: *Alicula* Schlechtendal, 1913); Storozhenko 1998: 97. Invalid name according to Article 39 of the Code (ICZN, 1999).

#### Diagnosis.

In Permulidae CuA is either simple or branching distal of its middle and CuA2 not individualized. In other families of the order Grylloblattida either the first fork of CuA is situated near the base, or CuA1 and CuA2 are well individualized.

#### Description.

 Medium-sized insects. Forewing uncolored, without spots and stripes. Costal area narrow to broad. SC ending near the apical third of wing or apically. RS originates in basal third or quarter of wing. The base of M and CuA distinctly separated or fused. M forked distinctly before or near the base of RS. MA simple or branched. MP desclerotized near the middle of wing. M5 not individualized. CuA not divided into CuA1 and CuA2, CuA is branched distal of its middle, with 2–4 branches, distal branches of CuA not parallel to posterior margin of wing, rare CuA simple. CuP simple, straight or gently curved. A1 simple. Cross-veins either simple or forming two or more rows of cells in the majority of areas. Hind wing similar to forewing but anal area enlarged. Body unknown.

#### Composition.

 Seven genera are known from the Permian of Europe and North America, two of them are described as new below.

#### Notes.

 The generic name *Alicula* Schlechtendal, 1913 (Insecta) is a junior homonym of *Alicula* Eichwald, 1830 (Mollusca). The name *Alicula* Schlechtendal, 1913 must be replaced by *Permula* Handlirsch 1919 (the next oldest available name from among its synonyms). Thus, the family name Aliculidae Storozhenko, 1997 must be replaced by a new replacement name Permulidae based on the valid name of the former type genus according to Article 39 of the Code (ICZN, 1999).

#### Key to genera of Permulidae

**Table:** 

1(10)	The basal portion of M distinctly separated from CuA.
2(5)	Costal area distinctly broader than subcostal one.
3(4)	The basal portion of costal area broadly rounded, without an abrupt narrowing	*Permula*
4(3)	Costal area distinctly narrowing in its basal portion	*Sojanopermula*
5(2)	Costal area narrower than or as broad as subcostal one.
6(7)	CuA simple	*Kazanalicula* gen. n.
7(6)	CuA with two branches.
8(9)	The base of anterior branch of CuA distinctly sclerotized	*Elmopterum*
9(8)	The base of anterior branch of CuA desclerotized	*Tshepanichoptera*
10(1)	The basal portion of M fused with CuA completely or at certain distance.
11(12)	First fork of CuA situated distinctly proximal to main fork of M. Costal area with a row of simple or bifurcated anterior branches of SC	*Mezenalicula* gen. n.
12(11)	First fork of CuA situated near to main fork of M. Anterior branches of SC form double row of cells near the middle of costal area	*Neprotembia*

### Permula

Handlirsch, 1919 nom. resurr.

http://species-id.net/wiki/Permula

Alicula Schlechtendal 1913: Pl. 2, Figs 10a, b (type species: *Alicula lebachensis* Schlechtendal, 1913, by monotypy); Carpenter 1992: 109 (partim); Storozhenko 1998: 97 (partim). Invalid name according to Article 56 of the Code (ICZN, 1999).
Permula Handlirsch 1919: 32 (type species: *Permula lebachensis* Handlirsch, 1919, by original designation); Handlirsch 1922: 77; Kukalová 1964: 46, 47 (partim).

#### Description.

 The base of costal area in forewing broadly rounded; costal area is considerably broader than subcostal one, crossed with simple or furcated anterior branches of SC; RA simple; RS with more than two branches; the base of M distinctly separated from CuA; M forked before the base of RS; CuA with 2–4 branches, first fork of CuA is situated proximal to fork of M; the base of anterior branch of CuA sclerotized; CuP curved or straight; A1 and A2 simple.

#### Composition.

 Four species from the Lower and Middle Permian of Europe.

#### Notes.

 Apparently being unaware of Schlechtendal’s paper, Handlirsch (1919) described *Permula lebachensis* as a new genus and species based on the same specimen as *Alicula lebachensis* Schlechtendal, 1913. Later Carpenter (1992) synonymized *Permula lebachensis* Handlirsch, 1919 with *Alicula lebachensis* Schlechtendal, 1913 and included *Permula acra* Kukalová, 1964 in the genus *Alicula*. But the generic name *Alicula* Schlechtendal, 1913 is a junior homonym of *Alicula* Eichwald, 1830 and must be replaced by *Permula* Handlirsch 1919. Herein two species are transferred from *Sojanopermula* to *Permula* based on the shape of costal area and one new species is described. Moreover, *Permula acra* Kukalová, 1964 is not congeneric with other species of *Permula* and is placed in a new genus of the family Ideliidae (see below).

#### Key to species

**Table:** 

1(4)	CuA with three or four branches.
2(3)	Anterior margin of wing straight; CuA with four branches	*Permula lebachensis*
3(2)	Anterior margin of wing convex; CuA with three branches	*Permula tshekardensis* comb. n.
4(1)	CuA with two branches.
5(6)	Cross-veins simple or forming a double row of cells in the areas between RA and RS, between MP and CuA, and between CuA and CuP	*Permula minor* comb. n.
6(5)	Cross-veins forming numerous rows of cells in all areas	*Permula edemskii* sp. n.

### Permula lebachensis

(Schlechtendal, 1913)

http://species-id.net/wiki/Permula_lebachensis

Fig. 1

Alicula lebachensis Schlechtendal 1913: Pl. 2, Figs 10a, b (holotype – fragment of the basal portion of forewing; drawing by Schlechtendal in Handlirsch 1906–1908: 348, tab. 34, fig. 34; depositary unknown; not studied); Carpenter 1992: 109; Storozhenko 1998: 97, fig. 181.
Permula lebachensis Handlirsch 1919: 32; Handlirsch 1922: 77; Kukalová 1964: 46, 47. Synonymized by Carpenter 1992: 109.

#### Horizon.

 Asselian Stage, Lebacher Schichten (the Lower Permian).

#### Locality.

 Lebach locality, Saar-Nahe Basin, Saarland, Germany.

#### Description.

 Anterior margin of forewing gently convex; costal area as broad as intercubital one; RS distinctly separated from MA; MP forked; CuA with four branches; CuP curved; cross-veins in almost all areas forming a row of two cells, cross-veins simple in the basal portion of areas between SC and R, and between M and CuA, cross-veins forming rows of three cells in the basal portion of intercubital area.

**Figures 1–7.**
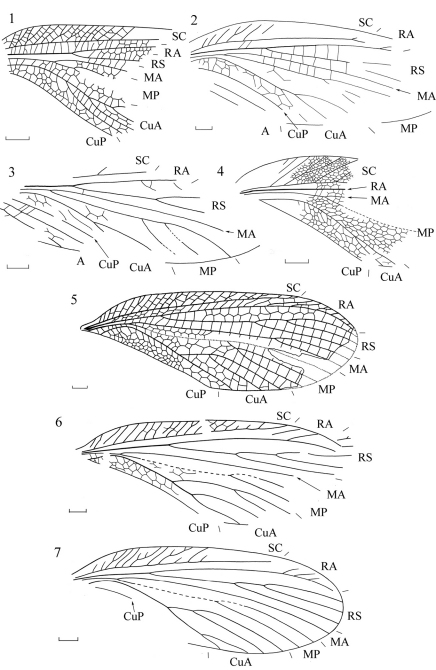
Forewings of Permulidae **1** *Permula lebachensis* (Schlechtendal) (after Handlirsch 1906–1908) **2** *Permula tshekardensis* Aristov (holotype PIN 1700/1151, after Aristov 2004) **3** *Permula minor* Aristov (holotype PIN 1700/4934, after Aristov 2004) **4** *Permula edemskii* sp. n. (holotype PIN 94/1027, orig.) **5** *Sojanopermula lucida* Storozhenko (holotype PIN 94/117, after Storozhenko 1992) **6** *Sojanopermula rasnitsyni* sp. n. (holotype PIN 117/301, orig.) **7** *Sojanopermula rasnitsyni* sp. n. (paratype PIN 117/195, orig.). Scale bars 2 mm.

#### Measurements.

 Length of forewing about 25 mm.

#### Notes.

*Permula lebachensis* Handlirsch 1919 is an objective synonym of *Alicula lebachensis* Schlechtendal 1913 because the descriptions of both species are based on the same specimen.

### Permula tshekardensis

(Aristov, 2004) comb. n.

http://species-id.net/wiki/Permula_tshekardensis

Fig. 2

Sojanopermula tshekardensis Aristov 2004a: 107, fig. 10a (holotype – positive imprint of incomplete forewing; PIN 1700/1151; examined).

#### Material.

 Holotype only.

#### Horizon.

 Kungurian Stage, Koshelevka Formation (the Lower Permian).

#### Locality.

 Tshekarda locality, Krasnaya Gora Mountain on the left bank of Sylva River near the Tshekarda village, Suksun District, Perm Region, Russia.

#### Description.

Anterior margin of forewing convex; SC reaching the apical quarter of wing; costal area distinctly broader than intercubital one; RS with more than two branches, distinctly separated from MA; MA simple or bifurcate near the apex of forewing; MP with three or four branches directed to the posterior margin of wing; CuA with three branches; CuP curved; A1 and A2 straight and simple; cross-veins are simple or forming a double row of cells in the areas between MP and CuA, and between CuA and CuP.

#### Measurements.

 Length of forewing 30 mm.

### Permula minor

(Aristov, 2004) comb. n.

http://species-id.net/wiki/Permula_minor

Fig. 3

Sojanopermula minor Aristov 2004a: 107, fig. 10b (holotype – positive and negative imprints of incomplete forewing; PIN 1700/4934; examined).

#### Material.

 Holotype only.

#### Horizon.

 Kungurian Stage, Koshelevka Formation (the Lower Permian).

#### Locality.

 Tshekarda locality, Krasnaya Gora Mountain on the left bank of Sylva River near the Tshekarda village, Suksun District, Perm Region, Russia.

#### Description.

RS with more than two branches, distinctly separated from MA; MA simple or probably with short terminal fork; MP with three branches sharply turning towards the posterior margin of wing; CuA with two branches; CuP straight; A1 and A2 simple and straight; cross-veins are simple or forming a double row of cells.

#### Measurements.

 Length of forewing about 23 mm.

### Permula edemskii

Aristov & Storozhenko sp. n.

urn:lsid:zoobank.org:act:7728E3EC-1FCE-409B-BF16-1C6A84D372AE

http://species-id.net/wiki/Permula_edemskii

Figs 4 15

#### Material.

 Holotype PIN 94/1027, positive and negative imprints of an incomplete forewing.

#### Type horizon.

 Kazanian Stage, Lower Kazanian Substage, Iva-Gora Beds (the Middle Permian).

#### Type locality.

 Soyana locality, right bank of the Soyana River 56–60 km upstream of the mouth, Mezen District, Arkhangelsk Region, Russia.

#### Description.

 Anterior margin of forewing distinctly convex; costal area considerably broader than intercubital one; R distinctly separated from M; CuA with two branches; CuP straight; cross-veins forming numerous rows of cells in all areas.

#### Measurements.

 Length of forewing about 30 mm.

#### Etymology.

 The new species is named in honor of the Russian geologist M.B. Edemsky, who collected the type specimen.

### Sojanopermula

Storozhenko, 1992

http://species-id.net/wiki/Sojanopermula

Sojanopermula Storozhenko 1992: 217 (type species: *Sojanopermula lucida* Storozhenko, 1992, by original designation); Storozhenko 1998: 98.

#### Description.

 The base of costal area in forewing distinctly narrowing; costal area is broader than subcostal one, crossed with simple or furcated anterior branches of SC; RA simple; RS originated in the basal quarter of the wing, with three branches; the base of M distinctly separated from CuA; M forked before the origin of RS; CuA with three or four branches, first fork of CuA is situated distinctly proximal to fork of M; the base of anterior branch of CuA sclerotized; CuP straight.

#### Composition.

 Two species from the Middle Permian of Europe.

#### Key to species

**Table:** 

1(2)	CuA with three branches. Costal area as broad as intercubital area	*Sojanopermula lucida*
2(1)	CuA with four branches. Costal area distinctly broader than intercubital one	*Sojanopermula rasnitsyni* sp. n.


### Sojanopermula lucida

Storozhenko, 1992

http://species-id.net/wiki/Sojanopermula_lucida

Fig. 5

Sojanopermula lucida Storozhenko 1992: 218, fig. 18 (holotype – positive imprint of forewing without apical part and anal area; PIN 94/117; examined); Storozhenko 1998: 98, fig. 184.

#### Material.

 Holotype only.

#### Horizon.

 Kazanian Stage, Lower Kazanian Substage, Iva-Gora Beds (the Middle Permian).

#### Locality.

 Soyana locality, right bank of the Soyana River 56–60 km upstream of the mouth, Mezen District, Arkhangelsk Region, Russia.

#### Description.

Anterior margin of forewing straight; SC reaching apical one fifth of the wing; costal area as broad as intercubital one; RS distinctly separated from MA; MA with fork; MP with three branches; CuA with three branches; cross-veins in the almost all areas simple, but forming a row of two cells in the area between RA and RS, in the basal portion of areas between MA, MP and CuA, and between branches of CuA; in the intercubital area cross-veins forming numerous rows of cells.

**Figures 8–14.**
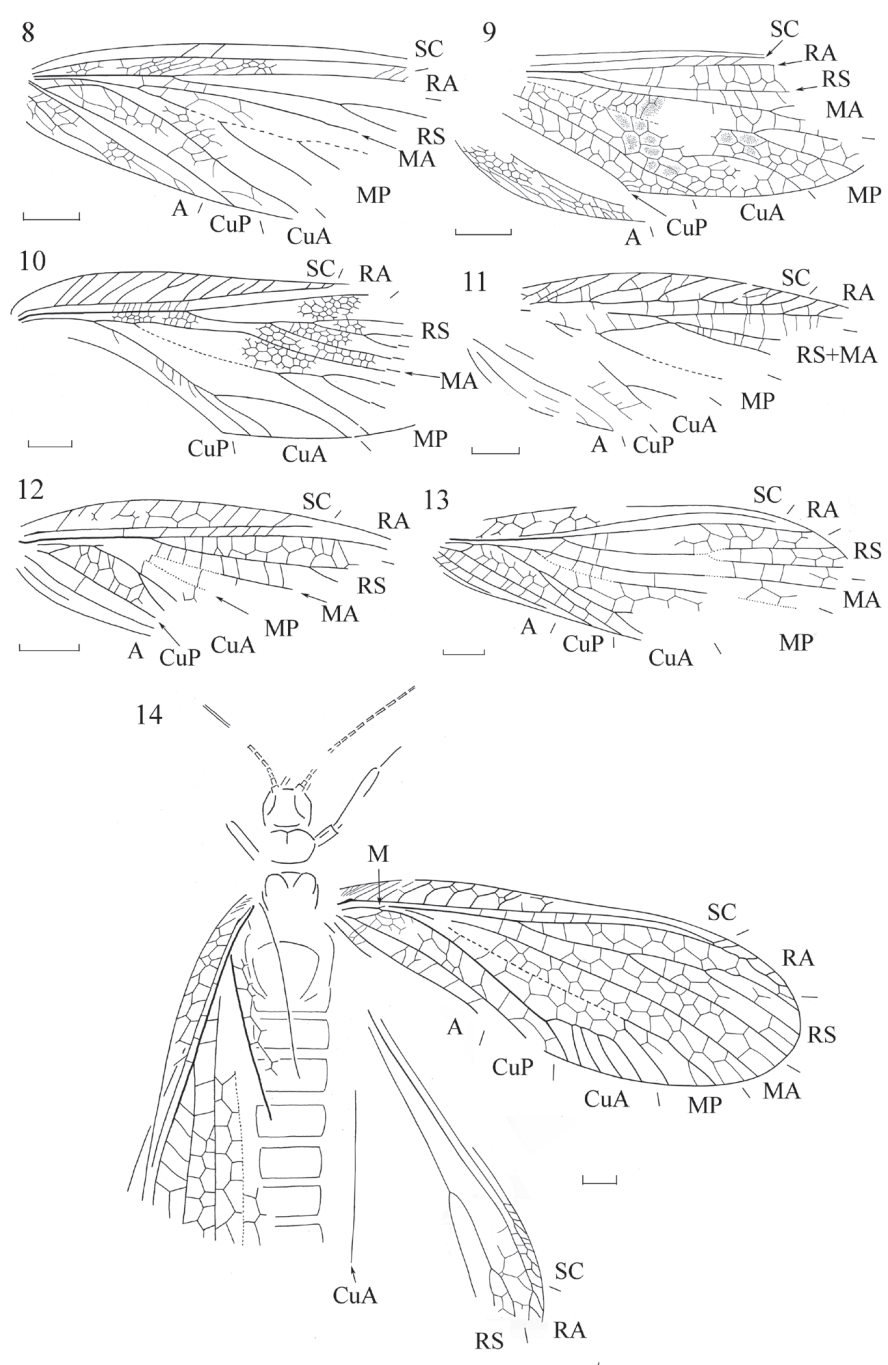
Forewings of Permulidae and Eoblattida incertae sedis **8** *Kazanalicula reducta* sp. n. (holotype PIN 117/467, orig.) **9** *Tshepanichoptera lacera* Aristov (holotype PIN 3286/14, after Aristov and Bashkuev 2008) **10** *Mezenalicula connata* sp. n. (holotype PIN 94/883, orig.) **11** *Mezenalicula conjuncta* sp. n. (holotype PIN 3840/1243, orig.) **12** *Neprotembia truncata* Aristov (holotype PIN 1700/1026, after Aristov 2004) **13** *Neprotembia complicata* Aristov (holotype PIN 1631/314, after Rasnitsyn et al. 2005) **14** *Neraphidia mitis* Novokshonov & Novokshonova (orig. reconstruction based on holotype PIN 1700/643 and spec. PIN 4987/10). Scale bars 2 mm.

#### Measurements.

 Length of forewing about 34.6 mm.

### Sojanopermula rasnitsyni

Aristov & Storozhenko sp. n.

urn:lsid:zoobank.org:act:7D76CEE0-2646-45F2-B738-072281C9CD4E

http://species-id.net/wiki/Sojanopermula_rasnitsyni

Figs 6, 7 16, 17

#### Material.

 Holotype PIN 117/301, positive imprint of incomplete forewing. Paratype PIN 117/195, positive imprint of forewing without anal area.

#### Type horizon.

 Kazanian Stage, Lower Kazanian Substage, Iva-Gora Beds (the Middle Permian).

#### Type locality.

 Soyana locality, right bank of the Soyana River 56–60 km upstream of the mouth, Mezen District, Arkhangelsk Region, Russia.

#### Description.

 Anterior margin of forewing straight; SC reaching apical quarter or one fifth of wing; costal area distinctly broader than intercubital area; RS separated from MA; MA simple; MP with two branches; CuA with four branches; cross-veins in the almost all areas simple, but forming a row of two cells in the basal portion of area between MP and CuA; in the intercubital area cross-veins forming two or three rows of cells.

**Figures 15–20.**
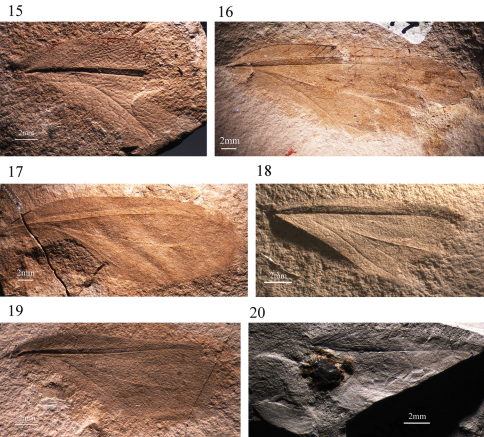
Forewings of Permulidae **15** *Permula edemskii* sp. n. (holotype PIN 94/1027) **16** *Sojanopermula rasnitsyni* sp. n. (holotype PIN 117/301) **17** *Sojanopermula rasnitsyni* sp. n. (paratype PIN 117/195) **18** *Kazanalicula reducta* sp. n. (holotype PIN 117/467) **19** *Mezenalicula connata* sp. n. (holotype PIN 94/883) **20** *Mezenalicula conjuncta* sp. n. (holotype PIN 3840/1243). Scale bars 2 mm.

#### Measurements.

 Length of forewing about 30 mm.

#### Etymology.

 The new species is named in honor of the Russian paleoentomologist Prof. A.P. Rasnitsyn.

### Kazanalicula

Aristov & Storozhenko gen. n.

urn:lsid:zoobank.org:act:2444F483-EB8B-4A2A-AD84-EA98E5B6E872

http://species-id.net/wiki/Kazanalicula

#### Type species.

*Kazanalicula reducta* Aristov & Storozhenko, sp. n.

#### Diagnosis.

 Similar to *Elmopterum* and *Tshepanichoptera* by the narrow costal area, but distinct from these in the simple CuA.

#### Description.

 The base of costal area in forewing gentle rounded; costal area is narrower than subcostal one, crossed with simple anterior branches of SC; RA simple; RS originated in the basal third of the wing, with two branches; the base of M distinctly separated from CuA; M forked slightly before the base of RS; CuA simple; CuP straight; A1 simple, A2 furcated.

#### Composition.

 One species from the Middle Permian of Europe.

#### Etymology.

 After the name of the Kazanian Stage and genus *Alicula*; gender feminine.

### Kazanalicula reducta

Aristov & Storozhenko sp. n.

urn:lsid:zoobank.org:act:5225F245-8B20-4FA3-93EE-5F39F7A019AB

http://species-id.net/wiki/Kazanalicula_reducta

Figs 8 18

#### Material.

 Holotype PIN 117/467, positive imprint of forewing without apical portion.

#### Type horizon.

 Kazanian Stage, Lower Kazanian Substage, Iva-Gora Beds (the Middle Permian).

#### Type locality.

 Soyana locality, right bank of the Soyana River 56–60 km upstream of the mouth, Mezen District, Arkhangelsk Region, Russia.

#### Description.

 Anterior margin of forewing weakly convex; costal area 2.5 times narrower than intercubital one; RS distinctly separated from MA; MA simple; MP with three branches directed to the posterior margin of wing; CuA simple; cross-veins in almost all areas forming a double row of cells.

#### Measurements.

 Length of forewing about 20 mm.

#### Etymology.

 From Latin *reductus* (distant).

### Elmopterum

Béthoux & Beckemeyer, 2007

http://species-id.net/wiki/Elmopterum

Elmopterum Béthoux and Beckemeyer 2007: 55 (type species: *Elmopterum rotundum* Béthoux & Beckemeyer, 2007, by monotypy); Aristov 2009: 37.

#### Description.

 In forewing costal area as broad as subcostal one, crossed with simple anterior branches of SC; RA simple; RS originated in the basal third of the wing, with three or four branches; M forked distinctly before the base of RS; CuA with two branches, fork of CuA is situated distinctly proximal to fork of M; the base of anterior branch of CuA sclerotized; CuP straight; several branches of anal veins reaching posterior wing margin. In hind wing RA simple, RS dichotomously branched, with four branches; M branched distal of the first fork of RS.

#### Composition.

 One species from the Lower Permian of North America.

### Elmopterum rotundum

Béthoux & Beckemeyer, 2007

http://species-id.net/wiki/Elmopterum_rotundum

Elmopterum rotundum Béthoux and Beckemeyer 2007: 55, fig. 5 (holotype – positive and negative imprints of an incomplete individual; MCZ 7468 and MCZ 7469; deposited in the Museum Comparative Zoology, Cambridge, USA; not examined).

#### Horizon.

 Leonardian Stage, Wellington Formation (the Lower Permian).

#### Locality.

 Elmo locality, Kansas, USA.

#### Description.

Anterior margin of forewing weakly convex; SC reaching the apical one fifth of the wing; costal area as broad as intercubital one; RS distinctly separated from MA; MA simple or with two branches directed to the apex of wing; MP with three branches directed to the posterior margin of wing; CuA with two branches; cross-veins in almost all areas forming a double row of cells.

#### Measurements.

 Length of forewing about 18 mm.

### Tshepanichoptera

Aristov, 2008

http://species-id.net/wiki/Tshepanichoptera

Tshepanichoptera Aristov in Aristov and Bashkuev 2008: 57 (type species: *Tshepanichoptera lacera* Aristov, 2008, by original designation).

#### Description.

 In forewing costal area narrow, as broad as subcostal one; anterior branches of SC absent; RA simple; RS originated in the basal third of the wing, simple; M forked distinctly basally to main fork of R; CuA with two branches, the fork of CuA is situated distinctly proximal to fork of M; the base of anterior branch of CuA desclerotized; CuP straight; A1 simple, A2 reduced.

#### Composition.

 One species from the Middle Permian of Europe.

### Tshepanichoptera lacera

Aristov, 2008

http://species-id.net/wiki/Tshepanichoptera_lacera

Fig. 9

Tshepanichoptera lacera Aristov in Aristov and Bashkuev 2008: 57, fig. 2d (holotype – positive and negative imprints of forewing without apex and with anal area separated; PIN 3286/14; examined).

#### Material.

 Holotype only.

#### Horizon.

 Urzhumian Stage (the Middle Permian).

#### Locality.

 Chepanikha locality, Rossokha River 1.8 km north of the Chepanikha village, Zavyalovsk District, Udmurtia, Russia.

#### Description.

 Anterior margin of forewing straight; SC reaching the apical quarter of wing; costal area considerably narrower than intercubital one; RS distinctly separated from MA; MA forked; MP with two branches directed to the posterior margin of forewing; CuA with two branches; cross-veins in almost all areas forming a double row of cells, but in the areas between MP and CuA, between branches of CuA and in anal area, cross-veins forming numerous rows of cells.

#### Measurements.

 Length of forewing about 18 mm.

### Mezenalicula

Aristov & Storozhenko gen. n.

urn:lsid:zoobank.org:act:B823B199-72D0-499E-A595-68097693F7E4

http://species-id.net/wiki/Mezenalicula

#### Type species.

*Mezenalicula connata* Aristov & Storozhenko, sp. n.

#### Diagnosis.

 Similar to *Neprotembia* by the base of M fused with CuA, but distinct from it in the more proximal first fork of CuA, and anterior branches of SC not forming double row of cells.

#### Description.

 The base of costal area in forewing broadly rounded; costal area broader than subcostal one, crossed with simple or furcated anterior branches of SC; RA simple; RS originated in the basal third or near the mid wing, with four or fewer branches; the base of M completely fused with CuA; M forked before the base of RS; CuA with two or three branches, first fork of CuA is situated distinctly proximal to fork of M; the base of anterior branch of CuA sclerotized; CuP straight; A1 and A2 simple.

#### Composition.

 Two species from the Middle and Upper Permian of Europe.

#### Etymology.

 After the name of the Mezen District in the Arkhangelsk Region of Russia and genus *Alicula*; gender feminine.

#### Key to species

**Table:** 

1(2)	Cross-veins forming a double row or numerous rows of cells. RS distinctly separated from MA	*Mezenalicula connata* sp. n.
2(1)	Cross-veins simple. RS fused with MA at short distance near the base	*Mezenalicula conjuncta* sp. n.

### Mezenalicula connata

Aristov & Storozhenko sp. n.

urn:lsid:zoobank.org:act:71A86E11-B0EC-4E75-AD48-255EF52A6030

http://species-id.net/wiki/Mezenalicula_connata

Figs 10 19

#### Material.

 Holotype PIN 94/883, positive imprint of incomplete forewing.

#### Type horizon.

 Kazanian Stage, Lower Kazanian Substage, Iva-Gora Beds (the Middle Permian).

#### Type locality.

 Soyana locality, right bank of the Soyana River 56–60 km upstream of the mouth, Mezen District, Arkhangelsk Region, Russia.

#### Description.

 Anterior margin of forewing convex in basal portion of wing; SC reaching the apical third of wing; costal area considerably broader than intercubital one; RS distinctly separated from MA; MA simple; MP with three branches directed to the posterior margin of forewing; CuA with three branches; cross-veins forming numerous rows of cells in all areas.

#### Measurements.

 Length of forewing about 21 mm.

#### Etymology.

 From Latin *connatus* (connate).

### Mezenalicula conjuncta

Aristov & Storozhenko sp. n.

urn:lsid:zoobank.org:act:4549C5ED-EB14-4526-A1F3-DA4DAB596428

http://species-id.net/wiki/Mezenalicula_conjuncta

Figs 11 20

#### Material.

 Holotype PIN 3840/1243, positive imprint of incomplete forewing.

#### Type horizon.

 Severodvinian Stage, Upper Severodvinian Substage, Poldars Formation, Kichuga Bundle (the Upper Permian).

#### Type locality.

 Isady locality, left bank of the Sukhona River 1.8 km downstream of the Mutovino near Isady village, Veliky Ustyug District, Vologda Region, Russia.

#### Description.

 Anterior margin of forewing convex in the mid wing; SC reaching the apical third of wing; costal area as broad as the intercubital one; RS fused with MA at short distance near the base; MA and MP simple; CuA with two branches; cross-veins in all areas simple.

#### Measurements.

 Length of forewing about 20 mm.

#### Etymology.

 From Latin *conjunctus* (connected).

### Neprotembia

Aristov, 2004

http://species-id.net/wiki/Neprotembia

Neprotembia Aristov 2004a: 105 (type species: *Neprotembia truncata* Aristov, 2004, by original designation).

#### Description.

 The base of costal area in forewing broadly rounded; costal area about two times broader than subcostal one; the anterior branches of SC form double row of cells near the mid of costal area; RA simple; RS originated in the basal third of the wing, with two branches; the base of M fused with CuA at certain distance; M forked before the base of RS; CuA with three branches, first fork of CuA is situated near to fork of M; the base of anterior branch of CuA sclerotized; CuP straight; A1 and A2 simple.

#### Composition.

 Two species from the Lower Permian of Europe.

#### Key to species

**Table:** 

1(2)	Anterior margin of forewing distinctly convex; the basal part of wing broader	*Neprotembia truncata*
2(1)	Anterior margin of forewing gently convex; the basal part of wing narrower	*Neprotembia complicata*

### Neprotembia truncata

Aristov, 2004

http://species-id.net/wiki/Neprotembia_truncata

Fig. 12

Neprotembia truncata Aristov 2004a: 105, fig. 9d (holotype – positive and negative imprints of incomplete forewing; PIN 1700/1026; examined).

#### Material.

 Holotype only.

#### Horizon.

 Kungurian Stage, Koshelevka Formation (the Lower Permian).

#### Locality.

 Tshekarda locality, Krasnaya Gora Mountain on the left bank of Sylva River near the Tshekarda village, Suksun District, Perm Region, Russia.

#### Description.

Anterior margin of forewing convex; SC reaching the apical quarter of wing; costal area as broad as intercubital area; RS distinctly separated from MA; MA and MP simple; CuA probably with three branches; cross-veins are simple in the subcostal area and forming double row of cells in the areas between RA and RS, and between CuA and CuP.

#### Measurements.

 Length of forewing about 15 mm.

### Neprotembia complicata

Aristov, 2005

http://species-id.net/wiki/Neprotembia_complicata

Fig. 13

Neprotembia complicata Aristov in Rasnitsyn et al. 2005: 69, Figs 1g, d (holotype – positive imprint of well preserved folded forewing; PIN 1631/314; examined).

#### Material.

 Holotype only.

#### Horizon.

 Kungurian Stage, Lek-Vorkuta Formation, Rudnik Subformation (the Lower Permian).

#### Locality.

 Khalmer-Yu coalfield, borehole KhK-371, deep 88 m, Pechora Basin, Perm Region, Russia.

#### Description.

Anterior margin of forewing weakly convex; SC almost reaching the wing tip; costal area as broad as intercubital one; RS distinctly separated from MA; MA and possibly MP are simple; CuA with three branches; cross-veins simple and forming double row of cells in the apical portion of the areas between RA, RS, MA, MP and CuA (in the subcostal area cross-veins absent).

#### Measurements.

 Length of forewing 13 mm.

##### Taxa erroneously included in Permulidae

**Order Grylloblattida Walker, 1914**

**Suborder Grylloblattina Walker, 1914**

**Family Ideliidae Zalessky, 1928**

### Acropermula

Aristov & Storozhenko en. n.

urn:lsid:zoobank.org:act:CA6C68E9-C9E6-4DC2-8A44-FF87AE4C48D2

http://species-id.net/wiki/Acropermula

#### Type species.

*Permula acra* Kukalová, 1964, Lower Permian of Czech Republic.

#### Diagnosis.

 By shape of forewing CuA, the new genus undoubtedly belongs to family Ideliidae. It is easily distinguished from all known genera of Ideliidae in excised base of costal area.

#### Description.

 Anterior margin of forewing strongly convex; costal area broad, crossed with mostly furcated anterior branches of SC, the base of costal area distinctly excised; subcostal area very narrow; RA simple; radial area broad, with numerous slightly backwardly slanted cross-veins; RS with several branches; M dividing before the origin of RS; CuA concavely bent, with a long pectinate series of branches. CuA2 not individualized; intercubital area with single intercalary branch of CuA; cross-veins forming numerous rows of cells in almost all areas.

#### Composition.

 A type species only.

#### Ethymology.

 After the species name *acra* and genus *Permula*; feminine in gender.

#### Notes.

 The description of a new genus is based on illustrations and diagnosis of *Permula acra* (Kukalová 1964).

### Acropermula acra

(Kukalová, 1964) comb. n.

http://species-id.net/wiki/Acropermula_acra

Permula acra Kukalová 1964: 47, fig. 2, tab. 1, fig. 1 (holotype – positive imprint of forewing, specimen no. 69/1963; deposited in the Department of Paleontology, Charles University, Prague; not studied).
Permula aera : Kukalová 1964: 47 (lapsus calami).
Alicula acra : Carpenter 1992: 109, fig. 64, 4; Storozhenko 1998: 98, fig. 182.

#### Horizon.

 Sakmarian Stage, Boskovice Graben, Letovice Formation (the Lower Permian).

#### Locality.

 Obora locality, 1 km north-west from the Obora village near Boskovice, Moravia, Czech Republic.

Metidelia **Martynov, 1937**

Metidelia asiatica **(Storozhenko, 1997)**

**Notes**. This species was described in the genus *Alicula* (Srorozhenko 1997). After examination of holotype it was transferred to the genus *Metidelia* of family Ideliidae by Aristov (2004b).

**Order Eoblattida Hanlirsch, 1906**

**Incertae familiae**

Neraphidia **Novokshonov & Novokshonova, 1997**

**Notes**. The genus *Neraphidia* was described in the grylloblattid family Protembiidae (Novokshonov and Novokshonova 1997), but later it was placed in family Aliculidae (Aristov 2004a). The examination of holotype of *Neraphidia mitis* Novokshonov & Novokshonova, 1997 (Fig. 14) allows us to eliminate this taxon from order Grylloblattida. *Neraphidia* is characterized by SC terminating on RA, and by M5 stronger than the main stems of M and CuA, both characters are typical for the order Eoblattida (in order Grylloblattida SC terminating on C or disappearing in the area between C and RA; M5 usually not individualized, if present in some families, when M5 is as broad as, or narrower than stems of M and CuA). Undoubtedly *Neraphidia* is similar to genera of the family Tillyardembiidae in the shape of SC and CuA, but distinguished from the latter by most basal fork of M (in Tillyardembiidae first fork of M is situated distinctly proximal). Herein *Neraphidia* is transferred to the order Eoblattida (sensu Aristov and Rasnitsyn 2009) as a genus of uncertain taxonomic position inside this order.

## Conclusion

The family Permulidae consists of thirteen species in seven genera. The oldset Lower Permian representative of the family, *Permula lebachensis*, is known from the Asselian of Central Europe. In the Leonardian Elmo locality (North America) Permulidae is represented by *Elmopterum rotundum* only. The Kungurian Chekarda locality (East Europe) is characterized by very rich fauna of Grylloblattida, but only three species of the family are described from here (*Permula tshekardensis*, *Permula minor* and *Neprotembia truncata*). Another, probably younger Kungurian representative of Permulidae, *Neprotembia complicata*,is known from the Pechora Basin (East Europe). Five Middle Permian species (*Permula edemskii* sp. n., *Sojanopermula lucida*, *Sojanopermula rasnitsyni* sp. n., *Kazanalicula reducta* gen. et sp. n. and *Mezenalicula connata* gen. et sp. n.) are described from the Kazanian Soyana locality in East Europe. A later Middle Permian species, *Tshepanichoptera lacera*, is known from Urzhumian Chepanikha locality (East Europe). The only Upper Permian representative of family, *Mezenalicula conjuncta* sp. n., is described from Severodvinian Isady locality in East Europe. Almost all species are known solely by their holotypes, and only *Sojanopermula rasnitsyni* sp. n. is known by two specimens. Thus, Permulidae was a widespread but not abundant and diverse Permian family of the order Grylloblattida.

## Supplementary Material

XML Treatment for Permulidae

XML Treatment for Permula

XML Treatment for Permula lebachensis

XML Treatment for Permula tshekardensis

XML Treatment for Permula minor

XML Treatment for Permula edemskii

XML Treatment for Sojanopermula

XML Treatment for Sojanopermula lucida

XML Treatment for Sojanopermula rasnitsyni

XML Treatment for Kazanalicula

XML Treatment for Kazanalicula reducta

XML Treatment for Elmopterum

XML Treatment for Elmopterum rotundum

XML Treatment for Tshepanichoptera

XML Treatment for Tshepanichoptera lacera

XML Treatment for Mezenalicula

XML Treatment for Mezenalicula connata

XML Treatment for Mezenalicula conjuncta

XML Treatment for Neprotembia

XML Treatment for Neprotembia truncata

XML Treatment for Neprotembia complicata

XML Treatment for Acropermula

XML Treatment for Acropermula acra
